# Supplementary material for: Predictors of the post-COVID condition following mild SARS-CoV-2 infection
Source: Nat Commun. 2023 Sep 20;14:5839. doi: 10.1038/s41467-023-41541-x (PMC10511472; doi:10.1038/s41467-023-41541-x)
Supplement: Supplementary file 1 — Supplementary Information [file 41467_2023_41541_MOESM1_ESM.pdf]

Supplementary information for

Predictors of the post-COVID condition following mild SARS-CoV-2 infection

B-A. Reme[1,2,*], J. Gjesvik[1,3] and K. Magnusson[1,4]

[1]Cluster for Health Services Research, Norwegian Institute of Public Health, Oslo, Norway.
[2]Department of Health Management and Health Economics, University of Oslo, Norway
[3]Section for Breast Cancer Screening, Cancer Registry of Norway, Norway.
[4]Clinical Epidemiology Unit, Orthopedics, Department of Clinical Sciences Lund, Lund University, Lund, Sweden.
*Corresponding author. Email: Bjorn-Atle.Reme@fhi.no

Table of contents

Supplementary Figure 1	2
Supplementary Table 1	3
Supplementary Figure 2	4
Supplementary Table 2	5
Supplementary Table 3	6
Supplementary Figure 3	7
Supplementary Figure 4	8
Supplementary Figure 5	9
Supplementary Figure 6	10
Supplementary Figure 7	11
Supplementary Figure 8	12
Supplementary Figure 9	13
Supplementary Figure 10	14

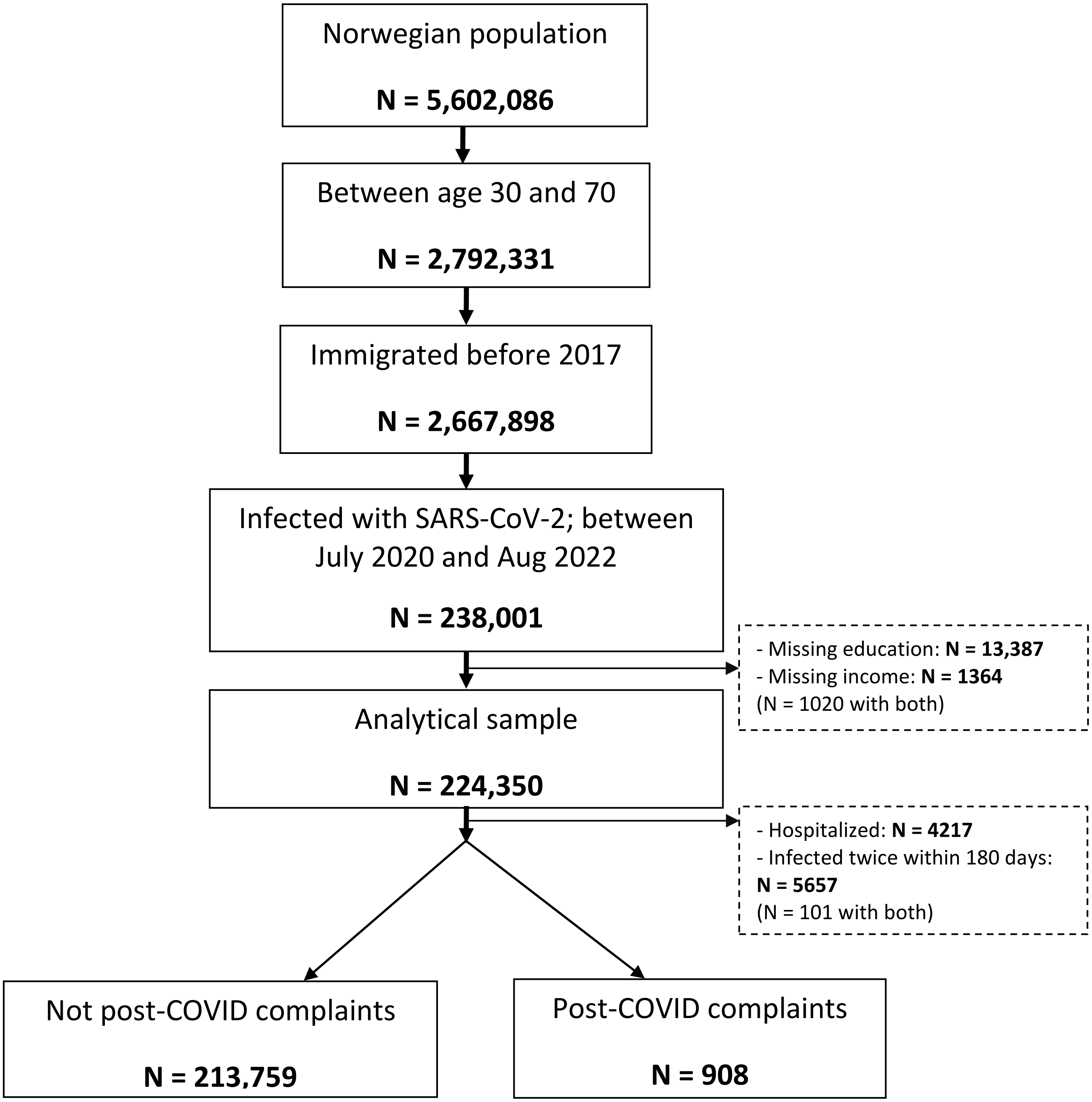

Supplementary Figure 1: Sample flow chart

Supplementary Table 1: Prevalence of conditions/complaints* used in concurrence with "R992" among individuals classified as post-COVID cases in the analytical sample

Description of health problem	ICPC-2 code	Number of observations	With registration also in 2017-2019
Pain (general/multisite and localized pain and symptoms from the musculoskeletal system, not classified elsewhere (neck, back, arms/hands, feet/legs))	A01, L01-L17, L18-L20, L29	95	56
Fatigue	A04, A05, A29	584	140
Cough	R05	39	7
Heart palpitations	K04, K05, K29	22	3
Shortness of breath	R02	167	8
Anxiety and depression	P03, P76, P01, P74	106	60
Brain fog (concentration or memory problems)	P20	36	1

*With condition/complaint we refer to all information that may be included in an ICPC-2 (International Classification of Primary Care 2) code: Diseases, disorders, signs, symptoms, and/or complaints as classified by the physician consulted.

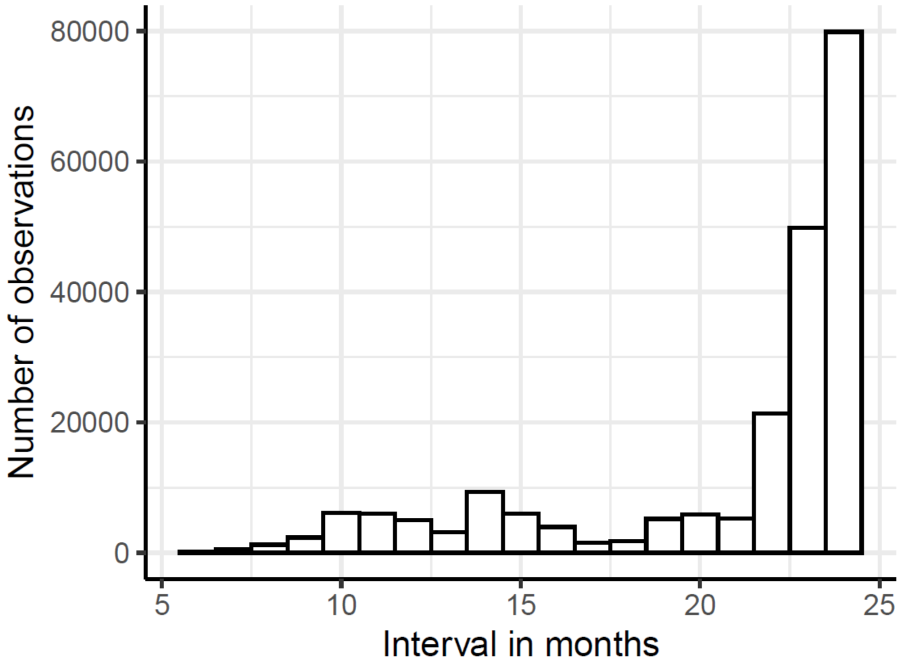

Supplementary Figure 2: Interval in months from observing pre-pandemic characteristics to infection in the analytical sample

The figure shows the distance in months between observing pre-pandemic characteristics and infection (x-axis) with the number of observations on the y-axis.

Supplementary Table 2: Descriptive statistics on covariates for different virus types

	**Virus type Mean/share (sd)**			
	**Wuhan**	**Alpha**	**Delta**	**Omicron**
Age	46.20 (10.42)	44.97 (9.78)	45.42 (10.10)	43.38 (9.23)
Income percentile	49.22 (28.33)	48.53 (28.40)	51.33 (28.85)	52.36 (28.95)
Education primary	0.24 (0.43)	0.27 (0.44)	0.19 (0.39)	0.18 (0.39)
Education secondary	0.36 (0.48)	0.35 (0.48)	0.35 (0.48)	0.33 (0.47)
Education lower university	0.28 (0.45)	0.26 (0.44)	0.31 (0.46)	0.32 (0.47)
Education higher university	0.12 (0.33)	0.12 (0.32)	0.15 (0.36)	0.17 (0.38)
Immigrant	0.40 (0.49)	0.41 (0.49)	0.30 (0.46)	0.29 (0.45)
**Health care utilization 2017-2019**				
General and Unspecified (A)	0.42 (1.67)	0.42 (1.57)	0.40 (1.69)	0.38 (1.58)
Blood and Immune Mechanism (B)	0.05 (0.61)	0.05 (0.59)	0.04 (0.58)	0.04 (0.53)
Digestive (D)	0.51 (1.76)	0.53 (1.91)	0.47 (1.80)	0.46 (1.72)
Eye (F)	0.09 (0.62)	0.09 (0.57)	0.08 (0.53)	0.08 (0.49)
Ear (H)	0.08 (0.59)	0.08 (0.65)	0.08 (0.62)	0.07 (0.57)
Cardiovascular (K)	0.50 (2.31)	0.43 (2.14)	0.44 (2.28)	0.33 (1.82)
Musculoskeletal (L)	2.32 (4.87)	2.29 (4.87)	1.99 (4.41)	1.82 (4.19)
Neurological (N)	0.34 (1.65)	0.37 (1.82)	0.33 (1.68)	0.32 (1.60)
Psychological (P)	1.13 (4.50)	1.19 (4.14)	1.13 (3.94)	1.10 (3.91)
Respiratory (R)	0.79 (1.97)	0.79 (1.97)	0.76 (2.03)	0.75 (1.88)
Skin (S)	0.39 (1.63)	0.38 (1.30)	0.39 (1.32)	0.38 (1.24)
Metabolic and Nutritional (T)	0.54 (2.14)	0.49 (2.07)	0.42 (1.88)	0.34 (1.68)

Note. For age and health care utilization average is reported. For the remaining, education and immigrant, share is reported.

Supplementary Table 3: Virus type, vaccination status at infection, and post-COVID condition incidence

Virus	Total (n)	Incidence	Incidence not vaccinated	Incidence vaccinated	Proportion vaccinated
Wuhan virus	22,945	1.27% [1.13%-1.42%]	1.27% [1.13%-1.42%]	NA	0.02%
Alpha virus	22,508	0.72% [0.61%-0.83%]	0.7% [0.59%-0.81%]	0.9% [0.43%-1.37%]	7%
Delta virus	78,518	0.44% [0.4%-0.49%]	0.48% [0.36%-0.59%]	0.43% [0.38%-0.48%]	82%
Omicron virus	90,696	0.12% [0.1%-0.14%]	0.06% [0%-0.12%]	0.12% [0.1%-0.15%]	93%

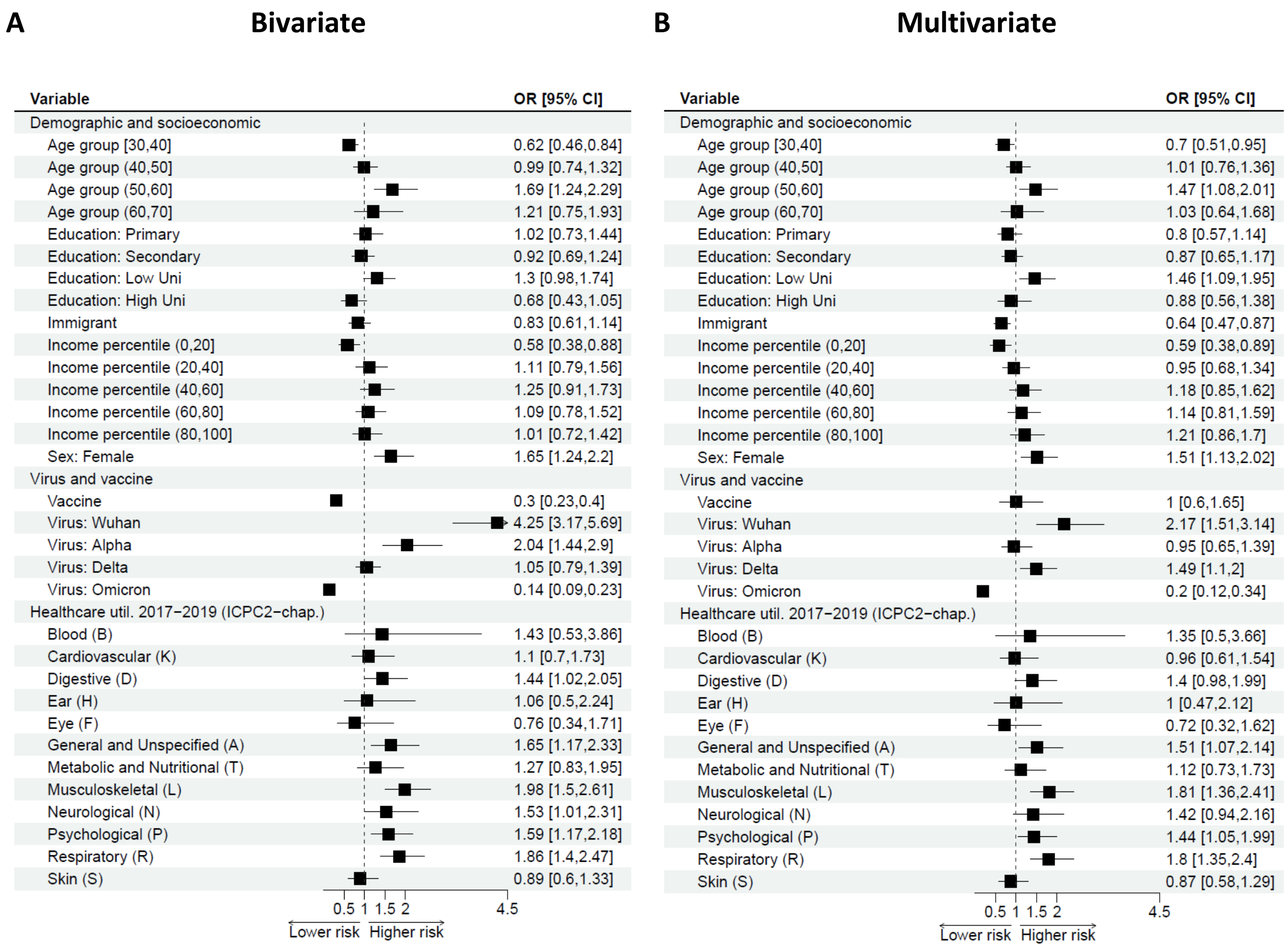

Supplementary Figure 3: Odds ratios for the post-COVID condition in bivariate (A) and multivariate (B) logistic regressions – post-COVID respiratory complaints

The squares show the estimated ORs for the post-COVID condition, with 95% confidence intervals represented by lines (n = 214,667 individuals). The post-COVID cases were limited to those reporting respiratory problems (ICPC2 codes: R02 and R05). Each predictor was included as a binary variable into the model, and the reference group (i.e., dashed vertical line, OR=1) for all predictors was "everyone else", i.e., everyone not having the predictor or characteristic of interest. Panel A shows the results from bivariate models, while panel B shows the results from multivariate models. Note that the multivariate models (B) used two sets of explanatory variables: for demographic characteristics, socioeconomic characteristics, virus type and vaccine status, the adjusted models accounted for health care utilization prior to the pandemic. While for health care utilization, the adjusted models accounted for demographic and socioeconomic factors, as well as vaccine status and virus type (see Table 3).

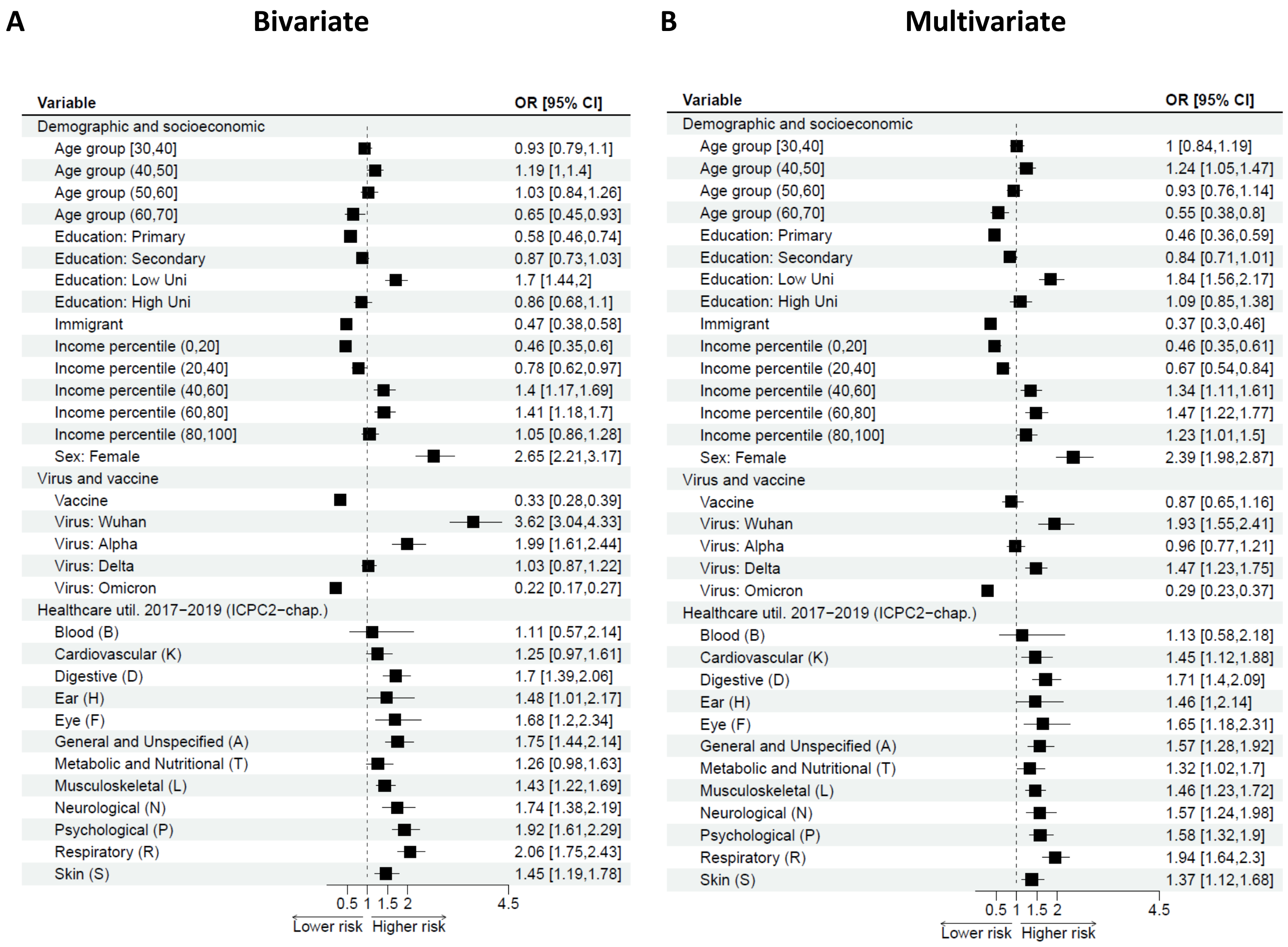

Supplementary Figure 4: Odds ratios for the post-COVID condition in bivariate (A) and multivariate (B) logistic regressions – post-COVID fatigue complaints

The squares show the estimated ORs for the post-COVID condition for each predictor, with 95% confidence intervals represented by lines (n = 214,667 individuals). The post-COVID cases were limited to those reporting fatigue (ICPC2 codes: A04, A05 and A29). Each predictor was included as a binary variable into the model, and the reference group (i.e., dashed vertical line, OR=1) for all predictors was “everyone else”, i.e., everyone not having the predictor or characteristic of interest. Panel A shows the results from bivariate models, while panel B shows the results from multivariate models. Note that the multivariate models (B) used two sets of explanatory variables: for demographic characteristics, socioeconomic characteristics, virus type and vaccine status, the adjusted models accounted for health care utilization prior to the pandemic. While for health care utilization, the adjusted models accounted for demographic and socioeconomic factors, as well as vaccine status and virus type (see Table 3).

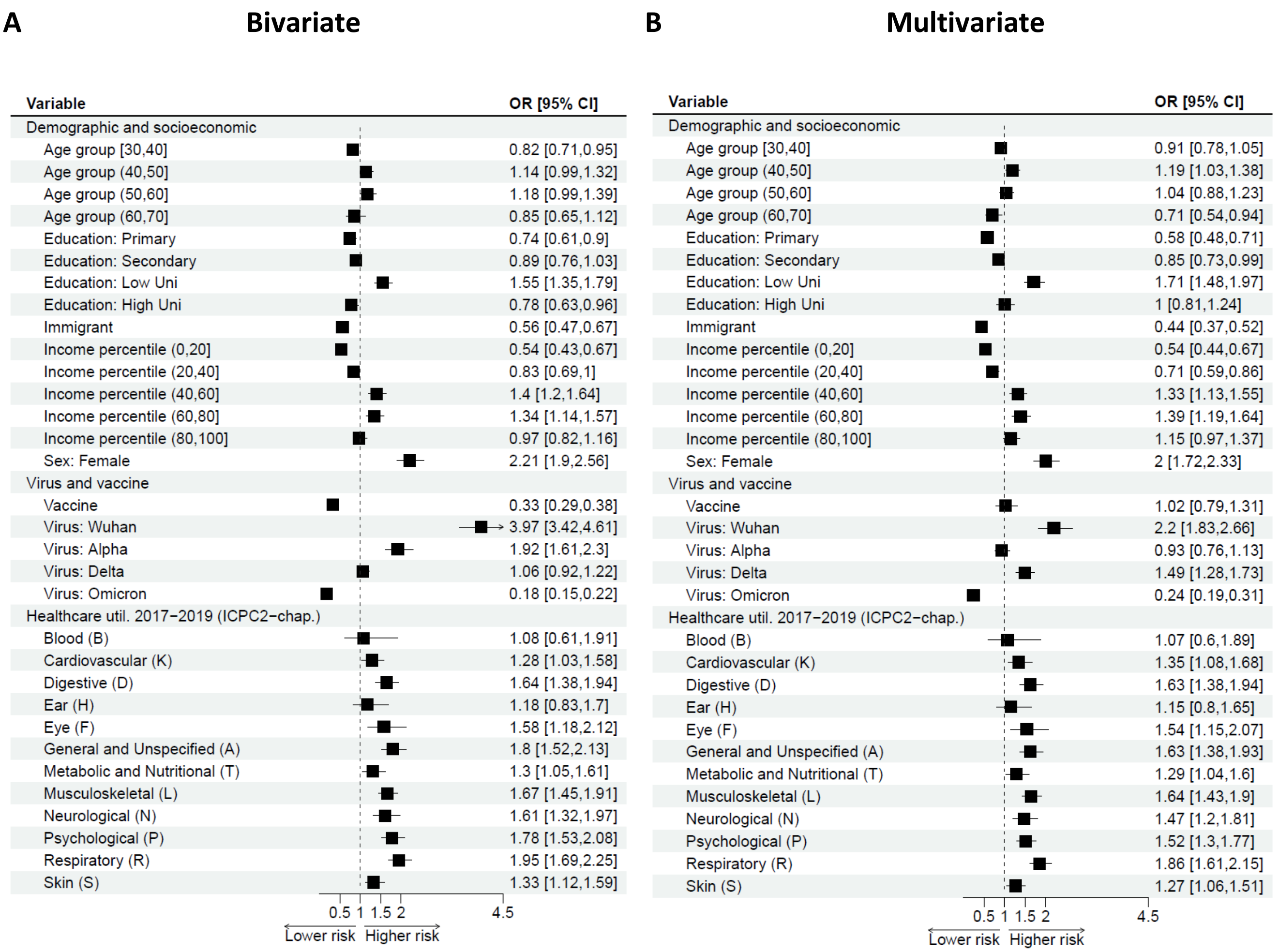

Supplementary Figure 5: Odds ratios for the post-COVID condition in bivariate (A) and multivariate (B) logistic regressions – recoding anxiety and depression post-COVID symptoms as non-post-COVID cases

The squares show the estimated ORs for the post-COVID condition, with 95% confidence intervals represented by lines (n = 214,667 individuals). The post-COVID cases were limited to those reporting symptoms other than anxiety and depression (cf. Table 1 for complete list). Each predictor was included as a binary variable into the model, and the reference group (i.e., dashed vertical line, OR=1) for all predictors was "everyone else", i.e., everyone not having the predictor or characteristic of interest. Panel A shows the results from bivariate models, while panel B shows the results from multivariate models. Note that the multivariate models (B) used two sets of explanatory variables: for demographic characteristics, socioeconomic characteristics, virus type and vaccine status, the adjusted models accounted for health care utilization prior to the pandemic. While for health care utilization, the adjusted models accounted for demographic and socioeconomic factors, as well as vaccine status and virus type (see Table 3).

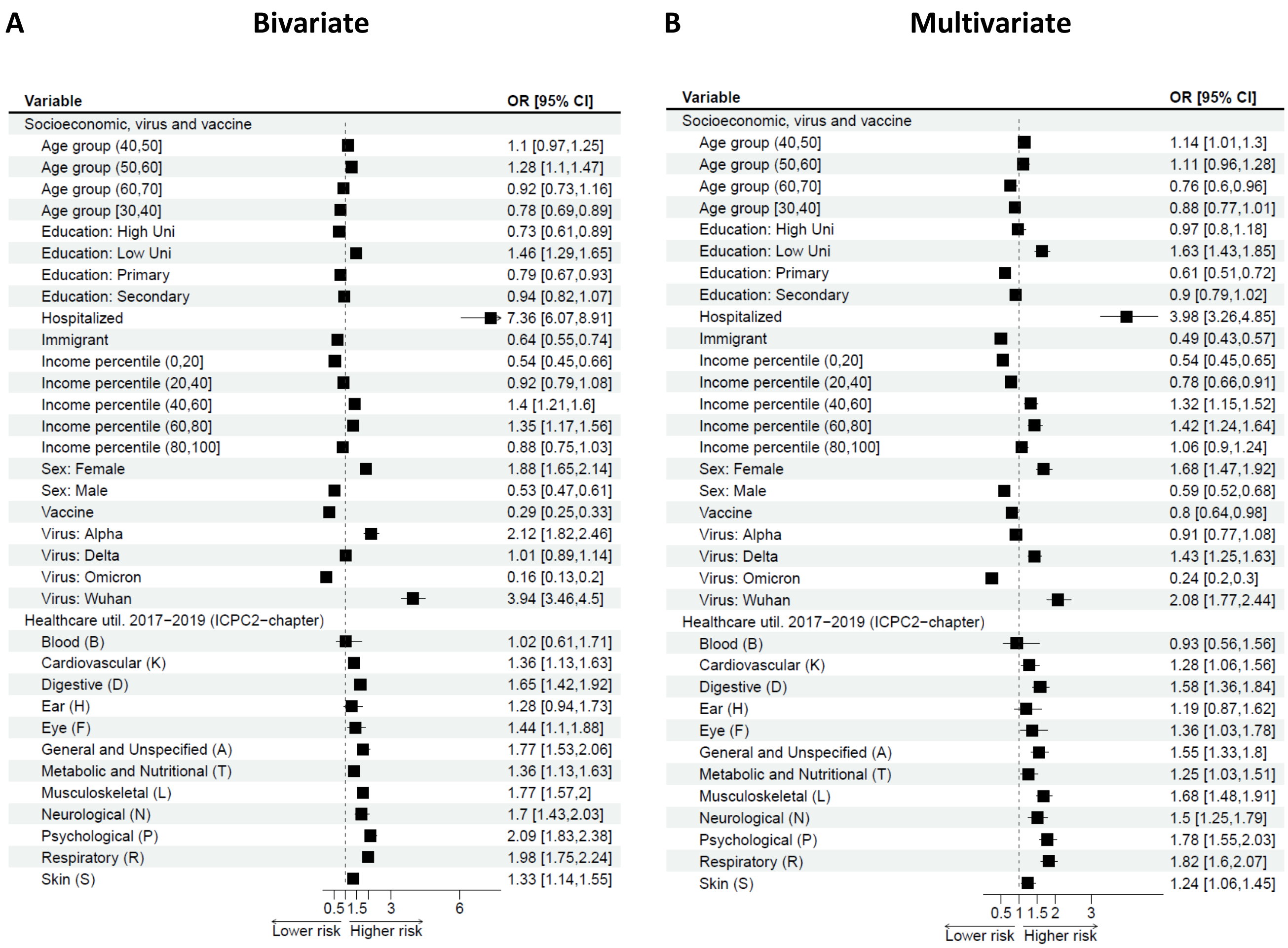

Supplementary Figure 6: Odds ratios for the post-COVID condition in bivariate (A) and multivariate (B) logistic regressions – including hospitalized.

The squares show the estimated ORs for the post-COVID condition, with 95% confidence intervals represented by lines (n = 218,693 individuals). Each predictor was included as a binary variable into the model, and the reference group (i.e. dashed vertical line, OR=1) for all predictors was "everyone else", i.e .everyone not having the predictor or characteristic of interest. Panel A shows the results from bivariate models, while panel B shows the results from multivariate models. Note that the multivariate models (B) used two sets of explanatory variables: for demographic characteristics, socioeconomic characteristics, virus type and vaccine status, the adjusted models accounted for health care utilization prior to the pandemic. While for health care utilization, the adjusted models accounted for demographic and socioeconomic factors, as well as vaccine status and virus type (see Table 3).

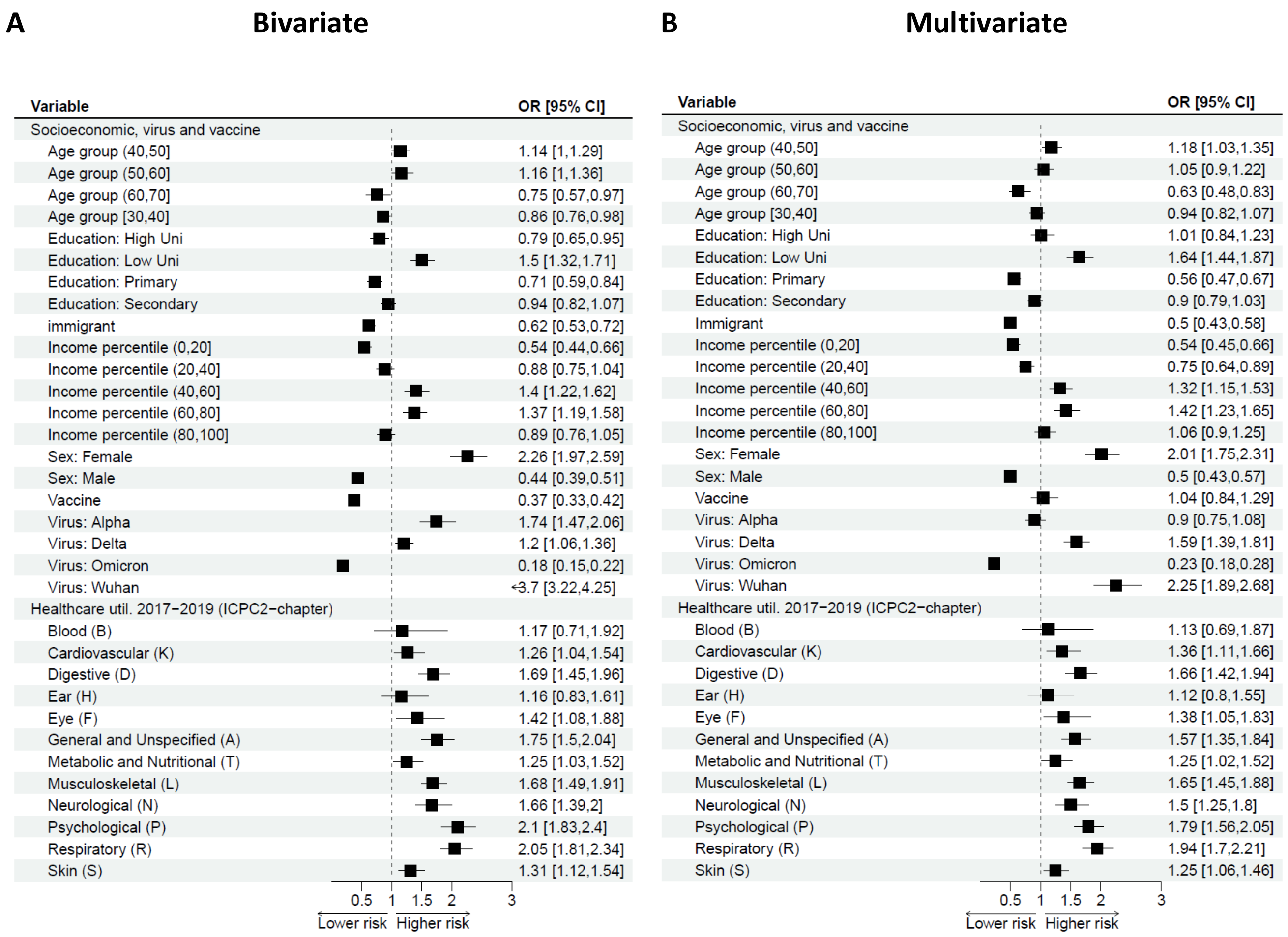

Supplementary Figure 7: Odds ratios for the post-COVID condition in bivariate (A) and multivariate (B) logistic regressions – including individuals infected within 180 days after first infection.

The squares show the estimated ORs for the post-COVID condition for each predictor, with 95% confidence intervals represented by lines (n = 220,223 individuals). Each predictor was included as a binary variable into the model, and the reference group (i.e., dashed vertical line, OR=1) for all predictors was "everyone else", i.e., everyone not having the predictor or characteristic of interest. Panel A shows the results from bivariate models, while panel B shows the results from multivariate models. Note that the multivariate models (B) used two sets of explanatory variables: for demographic characteristics, socioeconomic characteristics, virus type and vaccine status, the adjusted models accounted for health care utilization prior to the pandemic. While for health care utilization, the adjusted models accounted for demographic and socioeconomic factors, as well as vaccine status and virus type (see Table 3).

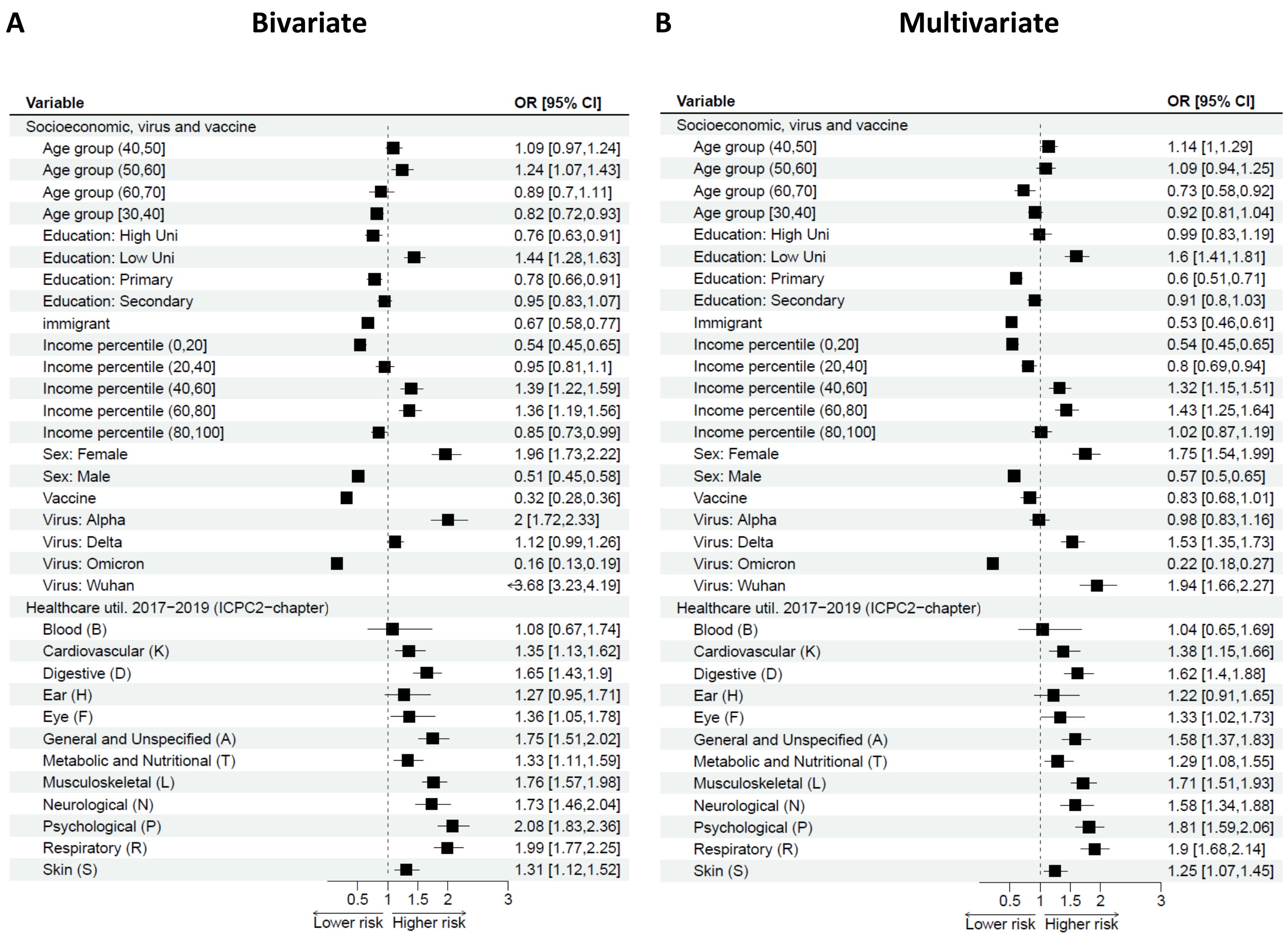

Supplementary Figure 8: Odds ratios for the post-COVID condition in bivariate (A) and multivariate (B) logistic regressions – including hospitalized individuals and individuals infected within 180 days after first infection.

The squares show the estimated ORs for the post-COVID, with 95% confidence intervals represented by lines (n = 224,350 individuals). Each predictor was included as a binary variable into the model, and the reference group (i.e., dashed vertical line, OR=1) for all predictors was “everyone else”, i.e., everyone not having the predictor or characteristic of interest. Panel A shows the results from bivariate models, while panel B shows the results from multivariate models. Note that the multivariate models (B) used two sets of explanatory variables: for demographic characteristics, socioeconomic characteristics, virus type and vaccine status, the adjusted models accounted for health care utilization prior to the pandemic. While for health care utilization, the adjusted models accounted for demographic and socioeconomic factors, as well as vaccine status and virus type (see Table 3).

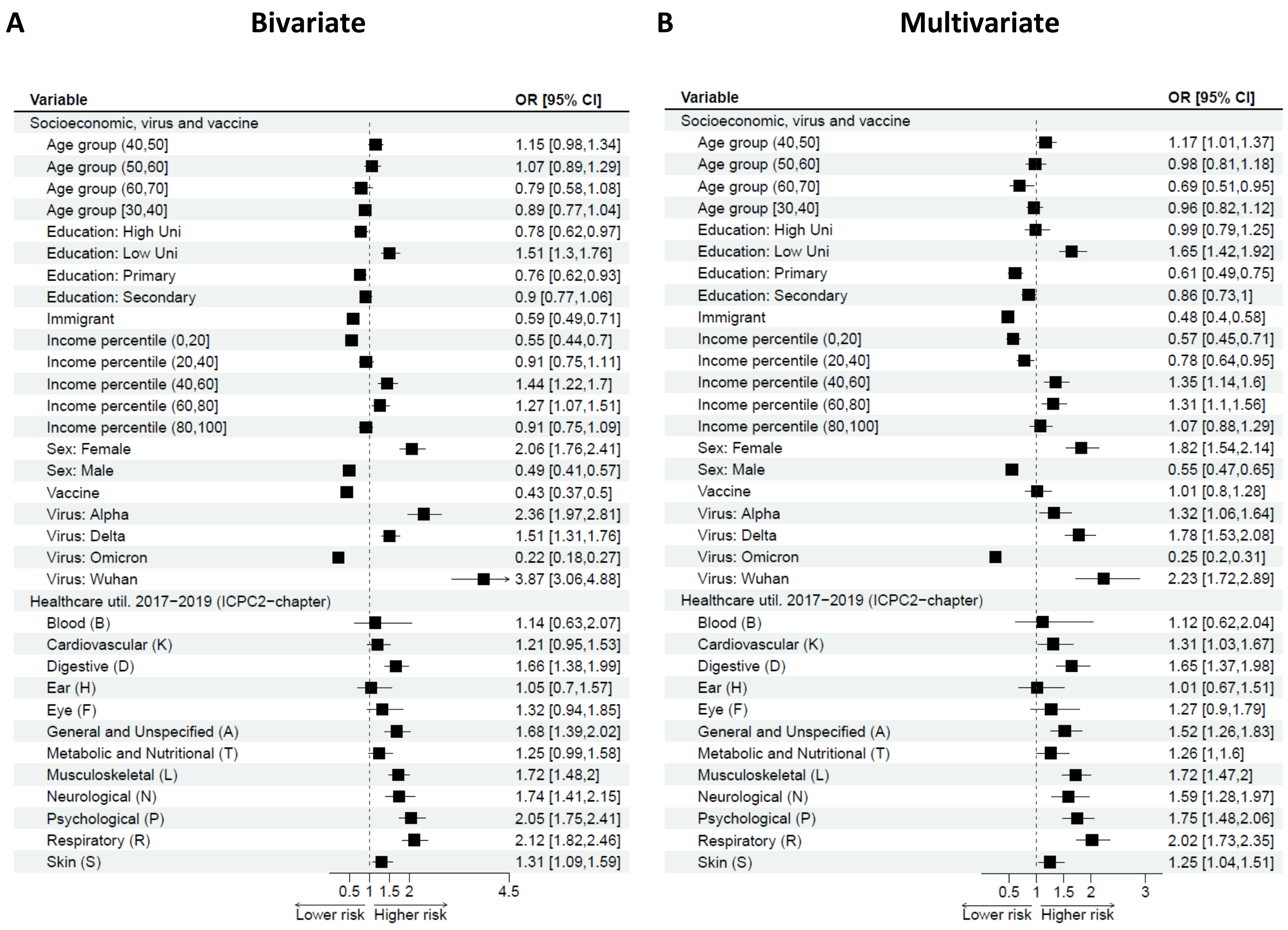

Supplementary Figure 9: Odds ratios for the post-COVID condition in bivariate (A) and multivariate (B) logistic regressions – starting the sample inclusion in January 2021.

The squares show the estimated ORs for the post-COVID condition, with 95% confidence intervals represented by lines (n = 198,304 individuals). Each predictor was included as a binary variable into the model, and the reference group (i.e., dashed vertical line, OR=1) for all predictors was “everyone else”, i.e., everyone not having the predictor or characteristic of interest. Panel A shows the results from bivariate models, while panel B shows the results from multivariate models. Note that the multivariate models (B) used two sets of explanatory variables: for demographic characteristics, socioeconomic characteristics, virus type and vaccine status, the adjusted models accounted for health care utilization prior to the pandemic. While for health care utilization, the adjusted models accounted for demographic and socioeconomic factors, as well as vaccine status and virus type (see Table 3).

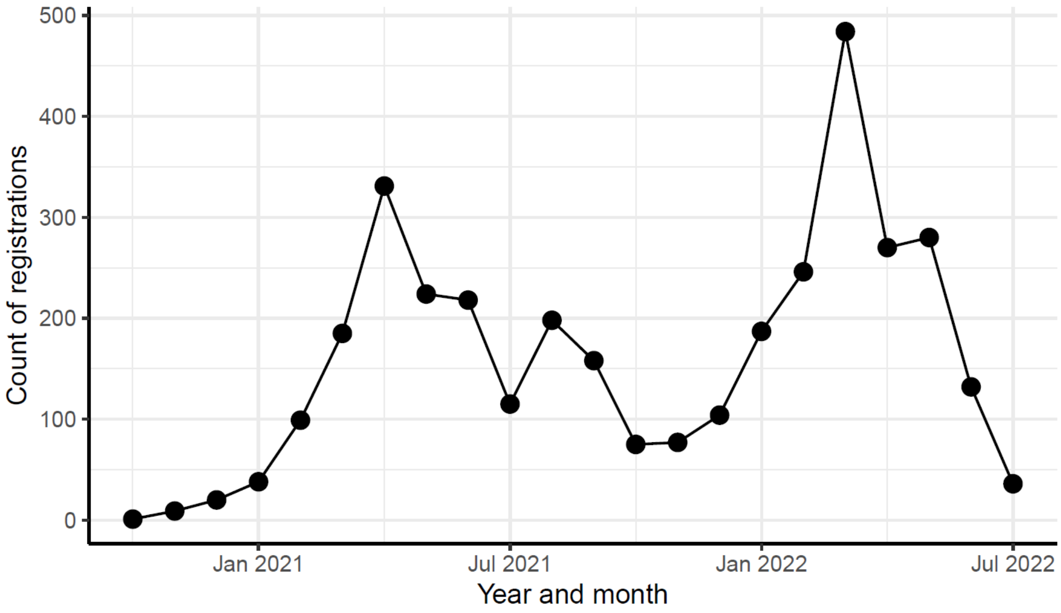

Supplementary Figure 10: Total count of number of main outcome registrations.

The figure shows the total count of registrations of post-COVID among individuals infected with SARS-CoV-2; between July 2020 and Aug 2022. A post-COVID registration was defined coded by an R992 code together with at least one of the following ICPC2-codes: Pain (A01, L01-L17, L18-L20, L29), Fatigue (A04, A05, A29), Cough (R05), Heart palpitations (K04, K05, K29), Shortness of breath (R02), Anxiety and depression (P03, P76, P01, P74), or Brain fog (P20).
