# Supplementary material for: Predictors of the post-COVID condition following mild SARS-CoV-2 infection
Source: Nat Commun. 2023 Sep 20;14:5839. doi: 10.1038/s41467-023-41541-x (PMC10511472; doi:10.1038/s41467-023-41541-x)
Supplement: Supplementary file 3 — Reporting Summary [file 41467_2023_41541_MOESM3_ESM.pdf]

nature portfolio

Corresponding author(s): Bjørn-Atle Reme
Last updated by author(s): Aug 21, 2023

Reporting Summary

Nature Portfolio wishes to improve the reproducibility of the work that we publish. This form provides structure for consistency and transparency in reporting. For further information on Nature Portfolio policies, see our Editorial Policies and the Editorial Policy Checklist.

Statistics

For all statistical analyses, confirm that the following items are present in the figure legend, table legend, main text, or Methods section.

n/a	Confirmed	
☐	☒	The exact sample size (*n*) for each experimental group/condition, given as a discrete number and unit of measurement
☐	☒	A statement on whether measurements were taken from distinct samples or whether the same sample was measured repeatedly
☒	☐	The statistical test(s) used AND whether they are one- or two-sided<br>*Only common tests should be described solely by name; describe more complex techniques in the Methods section.*
☐	☒	A description of all covariates tested
☒	☐	A description of any assumptions or corrections, such as tests of normality and adjustment for multiple comparisons
☐	☒	A full description of the statistical parameters including central tendency (e.g. means) or other basic estimates (e.g. regression coefficient) AND variation (e.g. standard deviation) or associated estimates of uncertainty (e.g. confidence intervals)
☒	☐	For null hypothesis testing, the test statistic (e.g. *F*, *t*, *r*) with confidence intervals, effect sizes, degrees of freedom and *P* value noted<br>*Give P values as exact values whenever suitable.*
☒	☐	For Bayesian analysis, information on the choice of priors and Markov chain Monte Carlo settings
☒	☐	For hierarchical and complex designs, identification of the appropriate level for tests and full reporting of outcomes
☒	☐	Estimates of effect sizes (e.g. Cohen's *d*, Pearson's *r*), indicating how they were calculated

Our web collection on statistics for biologists contains articles on many of the points above.

Software and code

Policy information about availability of computer code

Data collection	Data from the different administrative registers were stored in separate tables at a secure server at the Norwegian Institute of Public Health and were linked using a hashed personal ID number. The data from the different registers were linked in R using the RODBC-package (v.1.3-19).
Data analysis	All analyses were run in R (v.4.0.2), using the packages tidyverse (v.1.3.2), broom (v.1.0.2), tidymodels (v.0.1.4), ranger (v.0.13.1), and glmnet (v.4.1-3). Codes used to produce the results are provided here: Code used for producing the results presented in this study is available at https://github.com/remebjornatle/post_covid or https://zenodo.org/badge/latestdoi/661041995.

For manuscripts utilizing custom algorithms or software that are central to the research but not yet described in published literature, software must be made available to editors and reviewers. We strongly encourage code deposition in a community repository (e.g. GitHub). See the Nature Portfolio guidelines for submitting code & software for further information.

Data

Policy information about availability of data

All manuscripts must include a data availability statement. This statement should provide the following information, where applicable:

- Accession codes, unique identifiers, or web links for publicly available datasets
- A description of any restrictions on data availability
- For clinical datasets or third party data, please ensure that the statement adheres to our policy

The study was based on the Emergency Preparedness Register for COVID-19 (Beredt C19), a strictly regulated register available to selected authorized researchers in Norwegian Institute of Public Health. Sources included in the current study were the Norwegian Population Register (demographic characteristics), the Norwegian Tax Authorities and National Education Database (socioeconomic variables), the Norwegian Surveillance System of Communicable Diseases (results from all Polymerase Chain Reaction (PCR) testing), the Norwegian Immunization Registry (data on all vaccination against COVID-19), the Norway Control and Payment of Health Reimbursement Registry (primary health care visits before and during the pandemic) and the Norwegian Patient Registry (specialist health care visits before and during the pandemic).

The individual-level data that support the findings are thus not publicly available due to privacy laws. However, the data are accessible to authorized researchers after ethical approval and application to "helsedata.no/en" administered by the Norwegian Directorate of eHealth.¨The response time for data applications, following the necessary ethical approvals, varies by the demand and capacity at each register. It can range from months to two years, depending on the circumstances.

Human research participants

Policy information about studies involving human research participants and Sex and Gender in Research.

Reporting on sex and gender	We did not explicitly define what is meant by sex or gender in our article. The study includes both men and women, and sex differences in risk of post-COVID were estimated.
Population characteristics	All Norwegian residents, alive, and aged between 30 and 70 years old on Jan 1st, 2020, and who had their first positive SARS-CoV-2 PCR test, as registered in the Norwegian Surveillance System of Communicable Diseases, between July 1st 2020 and January 23rd 2022. Some exclusions were made in the main analysis based on missingness, hospitalization and reinfection between 90 and 180 days.
Recruitment	The study was based on national registers, hence it includes all official PCR tests performed by the healthcare system in Norway. Reporting test results were mandated by law.
Ethics oversight	The study was based on the emergency preparedness register for COVID-19 (Beredt C19), which was established to enable a quick and responsive way for the Norwegian government to gain knowledge related to the handling of the pandemic. The Ethics Committee of South-East Norway confirmed that external ethical board review was not required for studies deemed as within the purpose of the register (June 4, 2020, #153204). The Beredt 19 register is placed at The Norwegian Institute of Public Health, and this study was approved as within the purpose of the register.

Note that full information on the approval of the study protocol must also be provided in the manuscript.

Field-specific reporting

Please select the one below that is the best fit for your research. If you are not sure, read the appropriate sections before making your selection.

☒ Life sciences ☐ Behavioural & social sciences ☐ Ecological, evolutionary & environmental sciences

For a reference copy of the document with all sections, see nature.com/documents/nr-reporting-summary-flat.pdf

Life sciences study design

All studies must disclose on these points even when the disclosure is negative.

Sample size	The analytical sample had N = 214,667 observations/individuals. This sample size was the largest possible given the data access, after applying the following set of criteria: Norwegian residents, alive in the study period, aged between 30 and 70 years old on Jan 1st (2020), had their first positive SARS-CoV-2 PCR test between July 1st 2020 and January 23rd 2022, were not hospitalized due to Covid-19, were not reinfected within 90-180 days, and did not have missing data on education and/or income. See below for more details on data exclusions. No sample size calculation (power calculations) were performed.
Data exclusions	While N = 238,001 were eligible, the following exlusions were made: N = 13,651 were excluded either due to missingness on education and/or income. Then, N = 9683 were excluded either due to hospitalization due to Covid-19 or reinfection within 90-180 days. See TABLE for more details.
Replication	Replication tests in other samples were not performed. Several sensitivity tests were performed. These are all presented in the

Replication	supplementary materials and mentioned in the main text of the manuscript.<br><br>First, with regards to the sampling, the following sensitivity analyses were performed:<br>1) An analysis of risk factors when including hospitalized individuals,<br>2) an analysis of individuals with reinfection within 180 days,<br>3) an analysis of individuals either hospitalized and/or reinfected within 180 days,<br>4) an analysis of individuals who were infected after December 2020 (as opposed to the first period when the virus and its short- and long-term consequences were unknown).<br><br>Second, with regards to the outcome measure, the following additional analyses were performed using the the following secondary outcome measures:<br>1) Respiratory complaints (including cough and shortness of breath)<br>2) fatigue<br>3) when recoding individuals with anxiety and depression post-COVID symptoms as non-post-COVID case<br><br>The results across these sensitivity analysis naturally vary, but generally support our main findings.
Randomization	N/A, not an intervention study
Blinding	N/A, not an intervention study

Reporting for specific materials, systems and methods

We require information from authors about some types of materials, experimental systems and methods used in many studies. Here, indicate whether each material, system or method listed is relevant to your study. If you are not sure if a list item applies to your research, read the appropriate section before selecting a response.

Materials & experimental systems

n/a	Involved in the study
☒	☐ Antibodies
☒	☐ Eukaryotic cell lines
☒	☐ Palaeontology and archaeology
☒	☐ Animals and other organisms
☒	☐ Clinical data
☐	☒ Dual use research of concern

Methods

n/a	Involved in the study
☒	☐ ChIP-seq
☒	☐ Flow cytometry
☒	☐ MRI-based neuroimaging

Dual use research of concern

Policy information about dual use research of concern

Hazards

Could the accidental, deliberate or reckless misuse of agents or technologies generated in the work, or the application of information presented in the manuscript, pose a threat to:

No	Yes
☒	☐ Public health
☒	☐ National security
☒	☐ Crops and/or livestock
☒	☐ Ecosystems
☒	☐ Any other significant area

Experiments of concern

Does the work involve any of these experiments of concern:

No	Yes	
☒	☐	Demonstrate how to render a vaccine ineffective
☒	☐	Confer resistance to therapeutically useful antibiotics or antiviral agents
☒	☐	Enhance the virulence of a pathogen or render a nonpathogen virulent
☒	☐	Increase transmissibility of a pathogen
☒	☐	Alter the host range of a pathogen
☒	☐	Enable evasion of diagnostic/detection modalities
☒	☐	Enable the weaponization of a biological agent or toxin
☒	☐	Any other potentially harmful combination of experiments and agents
